# Association between medical student debt and choice of specialty: a 6-year retrospective study

**DOI:** 10.1186/s12909-019-1797-2

**Published:** 2019-10-28

**Authors:** Erik M. Fritz, Suzanne van den Hoogenhof, Jonathan P. Braman

**Affiliations:** 1 Department of Orthopaedic Surgery at the University of Minnesota in Minneapolis, 2450 Riverside Avenue South, Suite R200, Minneapolis, MN 55454 USA; 2 University of Minnesota Medical School in Minneapolis, Minneapolis, MN USA; 3 Department of Orthopaedic Surgery at the University of Minnesota in Minneapolis, Minneapolis, MN USA

**Keywords:** Student debt, Career choice, Medical school, Medical students

## Abstract

**Background:**

The effect of rapidly increasing student debt on medical students’ ultimate career plans is of particular interest to residency programs desiring to enhance recruitment, including primary care specialties. Previous survey studies of medical students indicate that amount of student debt influences choice of medical specialty. Research on this topic to date remains unclear, and few studies have included the average income of different specialties in analyses. The purpose of this study is to observe whether empirical data demonstrates an association between debt of graduating medical students and specialties into which students match.

**Methods:**

This was a retrospective cross-sectional study of a public institution including data from graduation years 2010–2015. For each included student, total educational debt at graduation and matched specialty were obtained. Average income of each specialty was also obtained. Statistical hypothesis testing was performed to analyze any differences in average debt among specialties; subanalysis was performed assessing debt for primary care (PC) versus non-primary care (NPC) specialties. Correlation between student debt and average specialty income was also evaluated.

**Results:**

One thousand three hundred ten students met the inclusion criteria and 178 were excluded for a final study population of 1132 (86%). The average debt was $182,590. Average debt was not significantly different among the different specialties (*P* = 0.576). There was no significant difference in average debt between PC and NPC specialties (PC $182,345 ± $64,457, NPC $182,868 ± $70,420, *P* = 0.342). There was no correlation between average specialty income and graduation debt (Spearman’s rho = 0.021, *P* = 0.482).

**Conclusions:**

At our institution, student indebtedness did not appear to affect matched medical specialty, and no correlation between debt and average specialty income was observed. Different subspecialties and residency programs interested in recruiting more students or increasing diversity may consider addressing alternative factors which may have a stronger influence on student choices.

## Background

Since the middle of the twentieth century, college tuition has increased at roughly twice the rate of general inflation [1] with one study citing over a 300% increase in the cost of public medical school in the final two decades [2]. Consequently, medical students need to utilize higher amounts of loans to finance their educations [3]. In 2015, more than 80% of graduating medical students had educational debt [4]; of those with debt, the median total was $183,000 [4].

However, it is unclear whether this increasing debt has implications on students’ career plans. Previous studies have investigated the relationship of graduate debt on specialty choice as this is of particular interest to primary care (PC) specialties, which are projected to have a shortage of 35,000 to 44,000 providers by 2025 [5]. Surveys of medical students report debt having an influence on specialty choice [6–11] while non-survey studies have demonstrated that debt appears to have little to no association with specialty [12–14]. Furthermore, few studies have included the average income of different specialties and the relationship this may have with debt.

To fill this gap, the present study provides an analysis of medical student debt, specialty choice, and average earnings per specialty over the past six years at our institution. The purposes of this study were to 1) determine whether an association exists between debt of graduating medical students and specialties into which students match, and 2) determine whether a correlation exists between indebtedness and average income of students’ chosen specialty.

## Methods

This was a retrospective cross-sectional study at the University of Minnesota Medical School, which is a public institution, including data from May 2010 to May 2015 utilizing the Strengthening the Reporting of Observational Studies in Epidemiology (STROBE) criteria. The Office of Student Finance provided total educational debt at graduation for each student, including both undergraduate and medical school debt. Specialty, determined from the residency match, was also obtained for each student. All personal data were de-identified prior to analysis; for further protection of privacy, specialties into which fewer than five students matched were grouped into the category, “Non-primary care: Unspecified.” Average income of each specialty for each graduation year was obtained from the Medical Group Management Association Physician Compensation and Production annual survey [15–20]. Values for debt and income from different years were adjusted to present day values utilizing a 3% annual inflation rate [21].

Every student who graduated from the institution’s Doctor of Medicine (M.D.) program from May 2010 to May 2015 was initially included in the study. Exclusion criteria included students for whom debt or match data were unavailable, students who did not match into an advanced or categorical program for a given year, and students who matched into a specialty into which compensation data was unavailable.

### Statistical methods

Statistical analysis was performed with SPSSⓇ Statistics Version 21.0 (IBM; Armonk, NY). A Kolmogorov-Smirnov test showed nonparametric distribution of all data; thus, any difference in debt between the specialties was evaluated by Kruskal-Wallis test. Spearman’s rho assessed correlation between average specialty income and graduation debt. Additionally, students were grouped into PC (family medicine, internal medicine, pediatrics, and combined internal medicine and pediatrics) and non-primary care (NPC) specialties; a Mann Whitney U test was used to evaluate differences in debt. Since physicians in pediatrics and internal medicine frequently subspecialize into higher-paying specialties, an additional subanalysis compared only family medicine versus NPC. To assess threshold effect, students were also grouped into $75,000 debt quintiles as previously described by Phillips et al. [9] and chi-square analysis evaluated whether more students went into PC at a particular debt quintile. For all analyses, *P* < 0.05 indicated a significant difference.

### Ethical approval

Ethical approval for this study was waived by the University of Minnesota Institutional Review Board; Study Number 1510E78929, date 10/30/2015. The IRB determined the study to be exempt from review under federal guidelines 45 CFR Part 46.101(b) category #4 EXISTING DATA; RECORDS REVIEW; PATHOLOGICAL SPECIMENS.

## Results

**Fig. 1:**
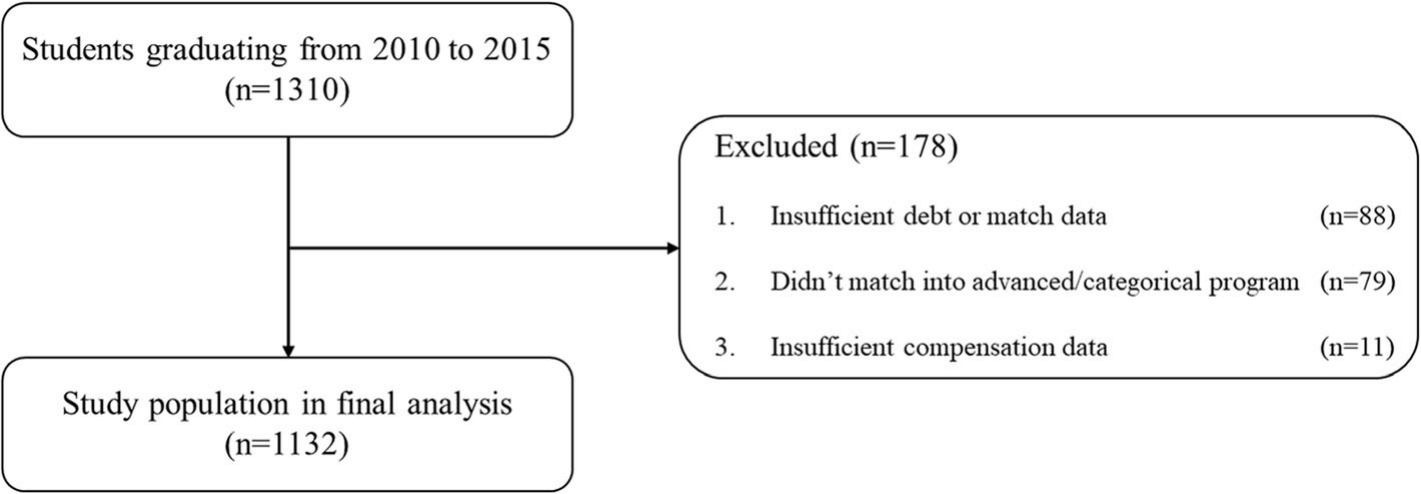
Flowchart demonstrating inclusion and exclusion criteria to obtain study population

In total, 1310 students met inclusion criteria. Of these, 178 students were removed after applying exclusion criteria, leaving a final population of 1132 (86%) (Fig. 1). The average debt was $182,590. Table 1 demonstrates a summary of the study population. The relative risk of matching into a nonprimary care specialty with a debt greater than the mean was 1.00 (95% confidence interval 0.02–50.40).

**Table 1:** Summary table of study population broken down by graduation year

Year	Total Students	Average Debt (USD)	Primary Care n (%)
2010	180	187,954	90 (50)
2011	202	182,701	117 (58)
2012	189	180,939	97 (51)
2013	177	183,766	90 (51)
2014	183	173,666	103 (56)
2015	201	186,320	104 (52)
Total	**1132**	**182,590**	**601 (53)**

**Fig. 2:**
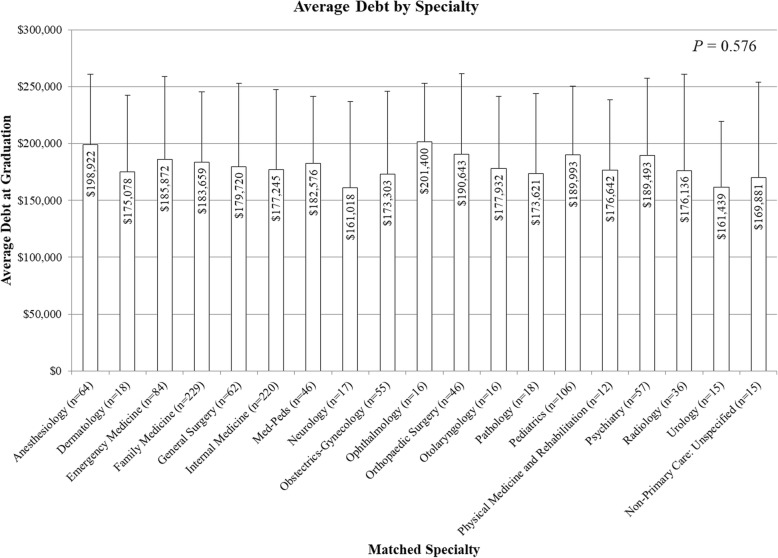
Graphical representation comparing graduating debt among the different specialties into which students matched. Debt is in 2015 dollars

**Fig. 3:**
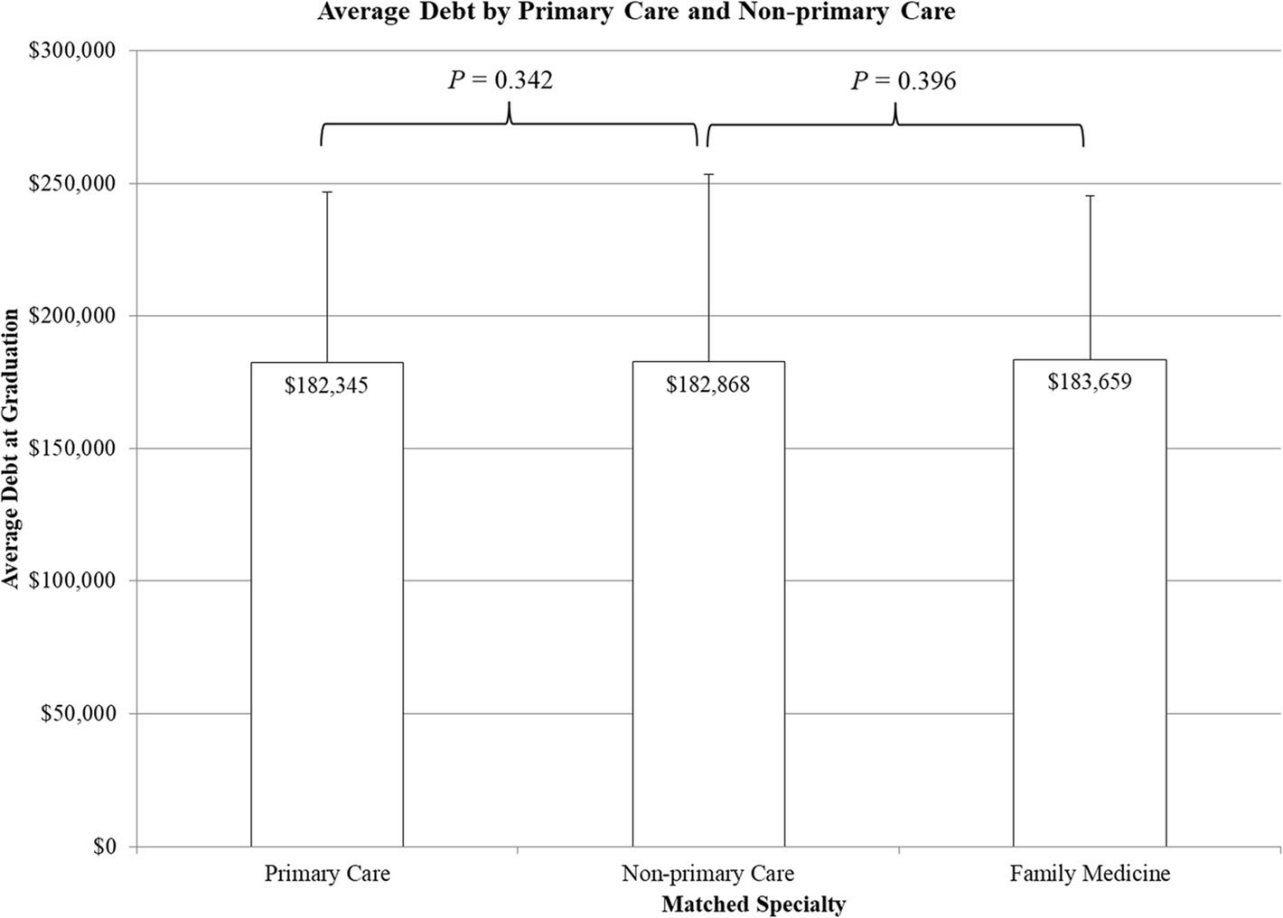
Graphical representation comparing graduating debt between the primary care and non-primary care specialties into which students matched. An additional subanalysis compares debt between students matching into family practice versus non-primary care specialties. Debt is in 2015 dollars

Amount of debt was not significantly different among different matched specialties (*P* = 0.576, Fig. 2). Furthermore, there was no significant difference in mean debt between PC and NPC specialties (PC $182,345 ± 64,457, NPC $182,868 ± $70,420, *P* = 0.342, Fig. 3). Subanalysis showed no significant difference in mean debt between students matching into family medicine versus NPC specialties (family medicine $183,569 ± $61,660, NPC $182,868 ± $70,420, *P* = 0.396, Fig. 3).

**Fig. 4:**
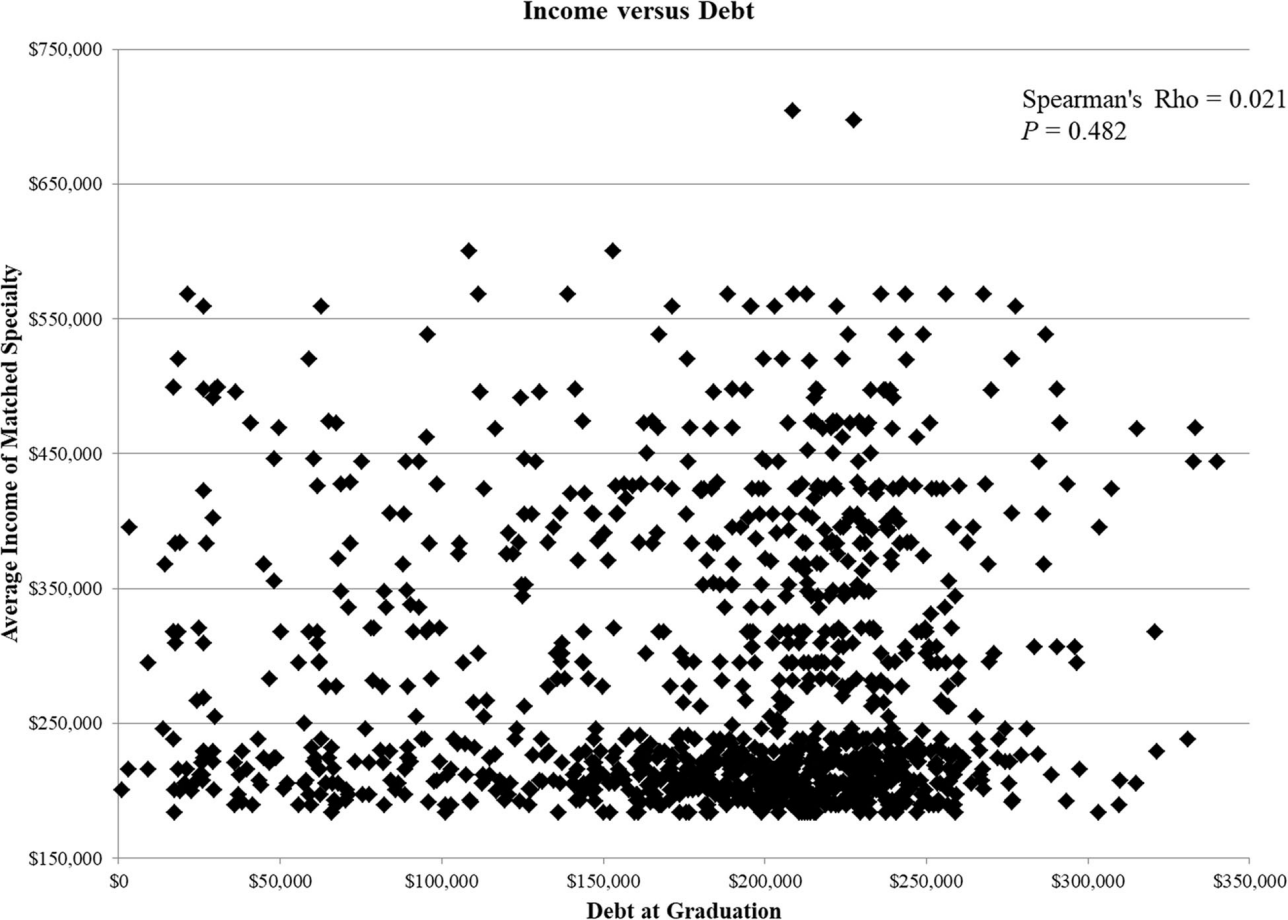
Scatterplot demonstrating average income versus student debt. Each datapoint indicates the debt with which the student graduated and the average income of his or her matched specialty from that particular graduation year. Debt and income are in 2015 dollars

Chi-square analysis showed no difference in students entering PC among different quintiles of debt (*P* = 0.112, Table 2). Finally, there was no correlation between average specialty income and graduation debt (Spearman’s rho = 0.021, *P* = 0.482, Fig. 4).

**Table 2:** Percentage of students matching into primary care versus non-primary care specialties stratified by range of student debt

Debt Range	Number of Students	Percent in Primary Care	Percent in Non-primary Care	
$0 - $74,999	125	52%	48%	
$75,000 - $149,000	163	47%	53%	
$150,000 - $224,999	523	57%	43%	
$225,000 - $299,000	308	50%	50%	
≥ $300,000	13	46%	54%	
				***P =*** **0.112**

## Discussion

The most important finding of this study is that student indebtedness at graduation did not have any association with choice of specialty. Moreover, there was no correlation between graduate debt and average income of the specialties into which students matched. This suggests that these factors appear independent even though the findings differ from numerous student survey studies [6–11].

Though medical student debt as it relates to career choice receives much interest in the published literature and media, few studies analyze empirical data obtained from offices of financial aid [12–14]. Instead, the majority of studies on this topic gather data through student surveys [6–11]. In 1993, Spar et al. [14] analyzed educational debt versus specialty of 1431 students who graduated between 1988 and 1990 from 6 private medical schools and found no relationship among levels of indebtedness and specialty preference [14]. By contrast, in 1996, Rosenthal et al. [13] performed a similar study analyzing 1350 students who graduated between 1987 and 1993 from Jefferson Medical College. They found that a high level of indebtedness (>$75,000) was a significant predictor of specialty choice away from family practice [13]. More recently, Kahn et al. [12] analyzed debt versus specialty of 2022 students who graduated between 2001 and 2005 from 3 medical schools; they found that graduates entering PC specialties did not have significantly differing debt compared to those entering other specialties, and debt was not a predictor of entering PC specialties [12]. Notably, for all three of these studies, data was obtained from the medical schools’ offices of financial aid [12–14]. In general, the main findings of our study are in agreement with these aforementioned results and demonstrate that the same trend appears today, even decades later; the Rosenthal study is the exception to this. While the reason for this difference can only be speculated upon, potential explanations may include the year, the location, private versus public medical school environment, or other unidentified factors. Future prospective multi-institutional studies may further assess these variables.

Most studies analyzing student debt versus specialty obtain data through student surveys [6–11]. Kassebaum and Szenas published articles in 1992 and 1993 [6, 7] evaluating the relationship between indebtedness and specialty choice among graduating medical students using data obtained from the annual American Association of Medical Colleges (AAMC) Medical School Graduation Questionnaire (GQ). In 1992, they found only 6.2% of students cited debt as having a “strong or major influence,” on specialty choice; this number nearly doubled in their 1993 study to 11.9% [6, 7]. Rosenblatt and Andrilla [11] did a similar study using the 2002 AAMC GQ; they found students with higher debt were significantly less likely to enter PC (debt > $150,000 odds ratio 0.94). In 2014 Phillips et al. [8] analyzed the AAMC GQ for graduates from 1988 through 2000. They found physicians graduating from public schools were more likely to practice PC at debt levels of $50,000 to $100,000 (2010 dollars); however at higher debt levels, probability of practicing PC decreased. Likelihood of practicing PC did not change with debt for private school graduates [8]. Philips and colleagues [9] performed a 2010 cross-sectional survey assessing students’ anticipated debt and specialty choice at three different medical schools from 2006 to 2008; when the group was analyzed as a whole, there was no relation between debt and specialty [9]. However, when stratifying family incomes, students from middle-income families anticipating more debt were less likely to plan PC careers [9]. Finally, Rohlfing et al. [10] performed an email survey of 102 medical schools with responses from 1846 students; results showed students with higher debt relative to their peers were more likely to choose a specialty with a higher income, were less likely to enter PC, and were less likely to practice in underserved locations [10].

Our present study and these previous studies [6–14] demonstrate a notable trend. The majority of studies with empirical data from offices of financial aid (including our present study) demonstrate no relation between debt and specialty. Yet, the majority of studies with student survey data demonstrate a consistent trend of debt correlating with specialty choice. This is an interesting discrepancy which may indicate that students’ perceptions of debt differ from reality; perhaps many students really believe that they cannot afford to enter primary care specialties with high indebtedness. In point of fact, Youngclaus and colleagues demonstrated that, even with very high amounts of debt, students can still affordably pursue primary care specialties [22]. Alternatively, perhaps students attribute debt as a justification for entering higher-paying specialties. Indeed, Dial and Haviland [23] argue that it should come as no surprise that the evidence has failed to establish a strong link between debt and specialty; the authors note that given the choice between a higher-paying and lower-paying specialty with similar responsibilities and working conditions, any student, regardless of debt level, would be more inclined to choose the higher paying specialty [23]. Moreover, Ebell published in both 1989 and 2008 two studies demonstrating a strong direct correlation between specialty incomes and residency program fill rates (1989 *r* = 0.85, 2008 *r* = 0.68 *P* = 0.03) [24, 25]. This may suggest that income has a much stronger influence on specialty choice than debt. Another possibility for the discrepancy may be the concept of a threshold effect in which debt does not influence career choice until a certain threshold is reached. In our present study, we observed no such threshold effect when the data was analyzed in debt quintiles.

### Limitations

There are several limitations to this study. First, we were unable to capture all debt data. Private student loans, auto loans, home loans, credit card debt, and other consumer debt were not factored into our calculations. Second, we were not able to perfectly capture career choice as some students did not match into their first-choice specialty; they may have dual-applied, gone through the supplemental offer and acceptance program, or matched in following years into a second-choice specialty. Moreover, our data does not capture plans for fellowship, which can substantially increase future income; however, we feel this limitation is addressed by our subanalysis comparing debt of those matching into family medicine versus NPC specialties. Finally, our study reflects data from a public university whereas different results may be obtained from other institutions with different student demographics, particularly private schools; this limits the generalizability of the study findings, though we feel the information can still be particularly helpful for other public institutions.

## Conclusion

At our institution, student indebtedness did not appear to affect matched medical specialty, and no correlation between debt and average specialty income was observed. Different subspecialties and residency programs interested in recruiting more students or increasing diversity may consider addressing alternative factors which may have a stronger influence on student choices.

## Abbreviations

NPC: Non-primary care
PC: Primary care
